# A novel method for large-scale culture of *Plasmodium falciparum* asexual blood stage and gametocytes in a Wave Bioreactor cell culture system

**DOI:** 10.1186/1475-2875-11-S1-P22

**Published:** 2012-10-15

**Authors:** Corine G Demanga, Jenny Eng, John P Dalton

**Affiliations:** 1 Institute of Parasitology, McGill University Ste Anne-de-Bellevue, Quebec, H9X 3V9, Canada

## 

The clinical manifestations of *Plasmodium falciparum* malaria occur when parasites are multiplying within erythrocytes. During this stage, some parasites develop into sexual forms (male and female gametocytes) that are transmitted to the mosquito during their feeding on blood. The development of new drugs or vaccines targeting both asexual and sexual blood stage parasites is important to strengthen existing tools for fighting against malaria, and this is an essential component of the overall strategy of malaria eradication.

However, there are still many unsolved questions concerning the cellular development and cell biology of *P.falciparum* asexual parasites, as well as for the onset and the development of the gametocyte stage. This is partly because of the inability to obtain good workable quantities of parasite material. Routine bulking-up of cultures in most laboratories involves establishing multiple culture flasks, which is very time consuming and labour intensive.

To obtain large quantities of parasite material with high consistency, we recently reported the use of the Wave Bioreactor system for the culture of asexual blood stage of *P. falciparum* in suspension [1]. Controlled wave-induced motion within the bioreactors provides low-shear, favourable hydrodynamic conditions and very efficient gas transfer that is ideal for parasite cultivation. We used the Wave Bioreactor™ 20/50 EHT which is composed of a single-use plastic Cellbag, the cultivation chamber, that sits on a heated and rocking platform. We established important parameters for maintaining *P. falciparum* cultures, such as rocking motion, temperature and gas flow. Moreover, by monitoring parasite growth along with glucose consumption, pH dynamic and lactic acid production we characterized the development of the parasites within the bioreactor. We showed that malaria parasites grow far better in these rocking cultures than in static flask cultures in terms of preserving parasite cell synchronicity and reducing the number of multiple infected erythrocytes. Finally, we established a simple and straightforward protocol for bulk cultures of up to 1L of culture of *P. falciparum* within five days.

To take advantage of the benefits of *P. falciparum* culture in suspension, we have now developed a simple protocol for gametocyte production and isolation from 1L culture in the wave bioreactor. This protocol includes 2 phases; the growth of the asexual parasites to stress conditions to induce gametocytes, followed by the maturation of gametocytes. In our experiments, approximately 8-10% of the asexual parasites commit to gametocytes. We reproducibly obtained mature gametocytes within 10 to 13 days (males in 10-12 days and females in 11 to 13 days). Batches of matures gametocytes can be collected from the same wave bag culture over several days. Finally, exflagellation tests confirm that gametocytes produced in the wave bioreactor are viable and, therefore, likely infectious for mosquitoes.

The use of the Wave Bioreactor is a breakthrough method that will allow large scale production of *Plasmodium falciparum* asexual blood stage and gametocytes for antigen or organelle isolation, high throughput screening of compound libraries, for infection of mosquitoes and for whole cell blood-stage malaria vaccine development under GMP compliant procedures.
